# *STK39* and *WNK1* Are Potential Hypertension Susceptibility Genes in the BELHYPGEN Cohort

**DOI:** 10.1097/MD.0000000000002968

**Published:** 2016-04-18

**Authors:** Alexandre Persu, Lucie Evenepoel, Yu Jin, Antonella Mendola, Gérard Ngueta, Wen-Yi Yang, Damien Gruson, Sandrine Horman, Jan A. Staessen, Miikka Vikkula

**Affiliations:** From the Pole of Cardiovascular Research (AP, LE, SH), Institut de Recherche Expérimentale et Clinique, Université catholique de Louvain, Brussels, Belgium; Cardiology Department (AP, GN), Cliniques Universitaires Saint-Luc, Université catholique de Louvain, Brussels, Belgium; Human Molecular Genetics (LE, AM, MV), de Duve Institute, Université catholique de Louvain, Brussels, Belgium; Studies Coordinating Centre (YJ, W-YY, JAS), Research Unit Hypertension and Cardiovascular Epidemiology, KU Leuven Department of Cardiovascular Sciences, University of Leuven, Leuven, Belgium; Population Health and Optimal Health Practices Research Unit (GN), CHU de Québec Research Centre, Québec, Canada; Pôle de recherche en Endocrinologie (DG), Diabète et Nutrition, Institut de Recherche Expérimentale et Clinique, Cliniques Universitaires Saint-Luc, Université catholique de Louvain, Brussels, Belgium; Department of Laboratory Medicine (DG), Cliniques Universitaires Saint-Luc, Université catholique de Louvain, Brussels, Belgium; and Research and Development VitaK Group (JAS), Maastricht University, Maastricht, The Netherlands.

## Abstract

The serine/threonine kinase With-No-Lysine (K) Kinase 1 (WNK1) activates the thiazide-sensitive Na^+^/Cl^−^ cotransporter through phosphorylation of STE20/SPS1-related proline/alanine-rich kinase, another serine/threonine kinase encoded by *STK39*. The aim of this study was to look for association between *WNK1* and *STK39* gene variants, and blood pressure (BP) and hypertension.

Seven hundred seventy-nine Caucasian hypertensive patients (HYP) recruited in 6 academic centers from Belgium, and 906 normotensive (NT) controls were genotyped for 5 single nucleotide polymorphisms—rs3754777, rs6749447, rs35929607 *(STK39*), rs1468326, and rs765250 (*WNK1*)—using the Snapshot method.

The rare TT genotype at the rs3754777 locus (*STK39*) was overrepresented in HYP versus NT (7.3% vs 3.0%, *P* = 0.0002). In the whole study population, the multivariable-adjusted odds ratio (OR) for having hypertension associated with the TT genotype was 5.9 (95% confidence interval: 2.2–15.6), and systolic BP was 10 mm Hg higher in TT compared with wild-type subjects (140.1 vs 130.4 mm Hg, *P* = 0.002). Similarly, the AA genotype at the rs1468326 locus (*WNK1*) was twice as frequent in HYP versus NT (5.5% vs 2.3%, *P* < 0.0001), and associated with an increased adjusted OR of hypertension (4.1; 1.5–11.7) and a higher systolic BP (139.8 vs 130.1 mm Hg, *P* = 0.003). In the whole cohort, a dose-dependent increase in systolic BP was observed according to the number of at-risk genotypes (0: 129.8 mm Hg; 1: 133.0 mm Hg; 2: 149.3 mm Hg, *P* = 0.02).

Single nucleotide polymorphisms rs3754777 (*STK39*) and rs1468326 (*WNK1*) were associated with hypertension and BP in our multicenter Belgian case-control study, which supports the role of *STK39* and *WNK1* as potential hypertension susceptibility genes. Replication in different clinical settings and study of other candidate loci belonging to the same molecular pathway is warranted.

## INTRODUCTION

Hypertension is a complex disease, influenced by multiple genetic and environmental factors. Heritability of blood pressure (BP) has been estimated to 30% to 55%.^1^ The loci so far identified account for only a small proportion of this heritability.^2^

A large body of evidence supports a crucial role for the kidney in BP regulation and pathogenesis of essential hypertension.^1,3^ In particular, several rare Mendelian forms of hypertension are due to mutations of genes involved in sodium and water reabsorption.^4,5^ Hence, it has been hypothesized that genetic variants affecting ion channels, transporters, and regulatory proteins involved in these pathways may be at the origin of more common forms of essential hypertension.

A genomewide association study (GWAS) performed in Amish, and then replicated in several non-Amish populations identified *STK39* as a possible hypertension susceptibility gene.^6^*STK39* encodes a serine/threonine kinase named STE20/SPS1-related proline/alanine-rich kinase (SPAK), which activates the thiazide-sensitive Na^+^/Cl^−^ cotransporter (NCC), expressed at the apical membrane of epithelial cells lining the distal convoluted tubule by phosphorylation of particular residues.^7–10^ In its turn, SPAK is activated through phosphorylation by the serine/threonine “With-No-Lysine (K) Kinase 1” (WNK1).^7–10^

In the current study, we took advantage of the BELHYPGEN cohort, including hypertensive patients (HYP) followed in tertiary referral centers from Belgium^11^ to look for an association between previously described genetic variants located within or in the vicinity of STK39 and WNK1, and hypertension and BP level.

## MATERIALS AND METHODS

### Population

All Caucasian patients with essential hypertension seen at the hypertension clinics of 6 Belgian University Hospitals (see BELHYPGEN consortium) from November 2005 to April 2010 were eligible. In all patients, a standardized Case Report Form (CRF) was completed, and blood samples were obtained for usual blood analysis and DNA extraction. In addition to main demographic characteristics, information was obtained on cardiovascular risk factors, target organ damage, previous cardiovascular events, number and classes of antihypertensive drugs, as well as lipid-lowering, antidiabetic, and antiplatelet medications. BP was measured after 5 minutes of rest in the sitting position according to the European Society of Hypertension guidelines.^12^ A mercury sphygmomanometer was used in 5 centers and a validated oscillometric device (Omron M4) in 1 center.

Controls were subjects with normal office BP (systolic BP <140 mm Hg and diastolic BP <90 mm Hg) in the absence of BP-lowering medications seen at the occupational health consultation of the Cliniques Universitaires Saint-Luc and Université catholique de Louvain (CESI, Louvain-en-Woluwé, Brussels). A simplified CRF and blood samples for DNA extraction were obtained in all participants.

Both CRF and DNA samples were anonimized. A unique ID number was used for both clinical and paraclinical information, and DNA. The study was approved by the Ethics Committees of the Cliniques Universitaires Saint-Luc and all other participating university centers. All participants gave written informed consent.

### DNA Extraction

Genomic DNA was extracted from leukocytes contained in the blood using the Wizard Genomic DNA Purification Kit (Promega, Madison, Wisconsin, USA). Briefly, the red blood cells, as well as the nuclei of the leukocytes, were lysed. Subsequently, proteins were precipitated, followed by the precipitation of DNA using isopropanol. The DNA pellet was washed with ethanol. Finally, DNA was rehydrated with the DNA Rehydration Solution.

### Genotyping

All subjects were genotyped for *STK39* rs3754777, rs6749447, and rs35929607, as well as for *WNK1* rs1468326 and rs765250. The different single nucleotide polymorphisms (SNPs) were sequenced using the SNaPshot minisequencing assay (Applied Biosystems, Foster city, California, USA), according to the manufacturer's instructions. Twenty nanograms of DNA were used for PCR amplifications that were performed using primers flanking the region containing the SNP of interest. Subsequently, amplicons were cleaned by FastAP Thermosensitive Alkaline Phosphatase (Fermentas, Waltham, Massachusetts, USA) and Exonuclease I treatment (Thermo Fisher Scientific, Waltham, Massachusetts, USA). A Forward or Reverse primer with a 3-prime end just at the SNP-site was used to perform a single-base extension reaction with fluorescently labeled ddNTPs. The products were purified with the FastAP, denatured using the Hi-Di Formamide, and run on an ABI3130xl Genetic Analyzer (Applied Biosystems). The analysis of the sequences was performed using the GeneMapper Software (Applied Biosystems), which shows peaks of different colors, reflecting the genotypes of the SNPs. Primers and conditions are available on request.

### Statistical Analysis

We used SAS, version 9.3, for database management and statistical analysis; we compared means and proportions, using ANOVA and the χ^2^ statistic, respectively. We tested the Hardy-Weinberg equilibrium using the SAS procedure PROC ALLELE. We included in our models covariables with known physiologic relevance for hypertension. We additionally searched for possible covariables, using stepwise multiple regression with *P* values for independent variables to enter and stay in the models set at 0.15. For studies with hypertension as outcome, we computed odds ratios (ORs) using logistic regression model. In multivariable-adjusted analyses, we computed OR adjusted for sex, age, body mass index (BMI), and total number of antihypertensive drug classes. We considered additive (BB vs AB vs AA) and recessive (BB vs AB + AA) genetic models.

## RESULTS

### Descriptive Analysis

The main characteristics of HYP and normotensive (NT) controls are shown in Table 1. Characteristics specifically collected in HYP but not in NT subjects are shown in Table 2. Most HYP initially had moderate-to-severe hypertension, and were relatively well controlled under a median of 2 antihypertensive drug classes. Compared with controls, hypertensive subjects had 30/16 mm Hg higher average BP despite antihypertensive treatment, they were 20 years older, had significantly higher BMI, and reported more frequently a familial history of early hypertension and cardiovascular disease. In contrast with HYP, no participant from the control group had previous or current cardiovascular disease and only 84 (0.6%) reported diabetes mellitus.

**TABLE 1:**
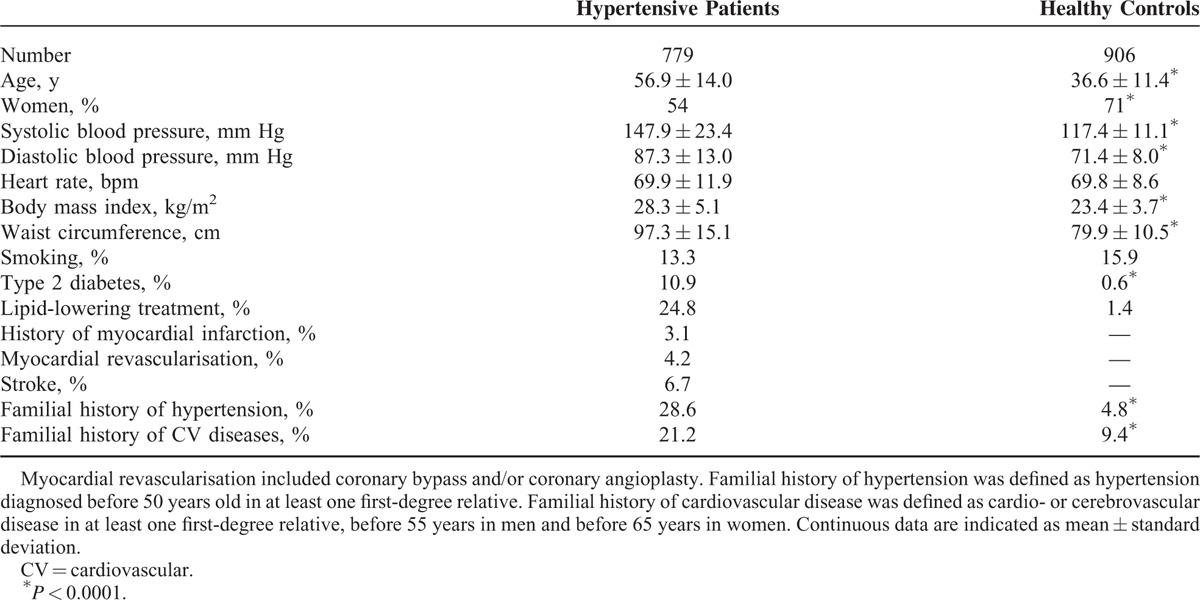
Main Characteristics of Cases and Controls Included in the BELHYPGEN Study

**TABLE 2:**
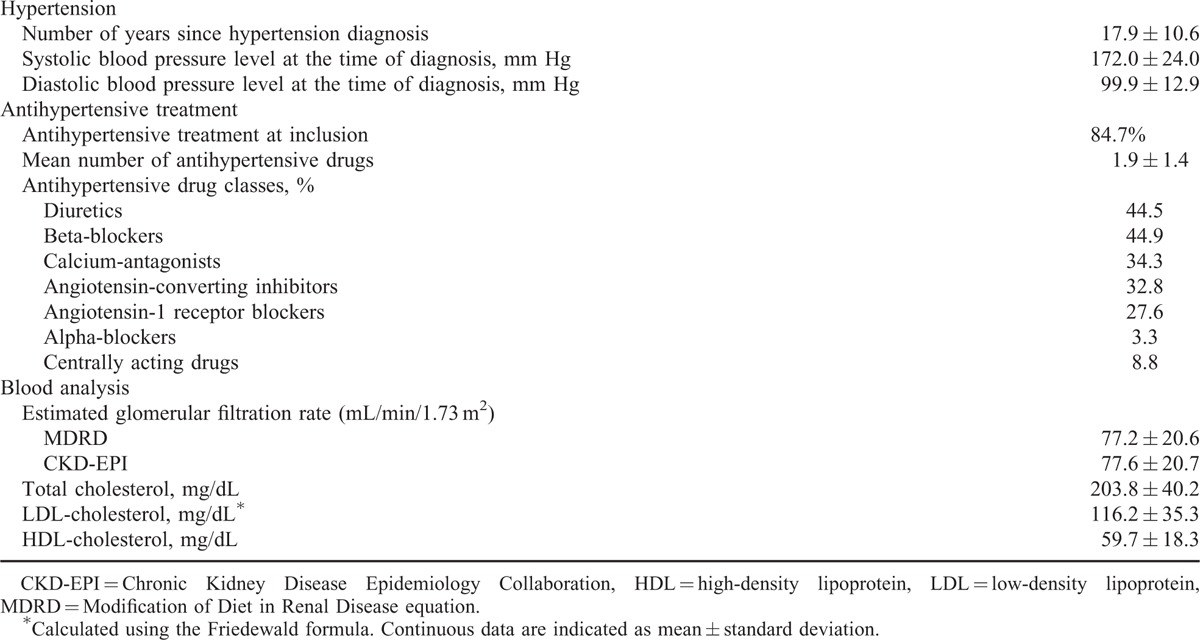
Additional Characteristics of Hypertensive Patients Included in BELHYPGEN Study

Variants rs3754777 and rs1468326 were strongly associated with hypertension. A borderline significant association was also found for rs7765250. The distribution of genotypes for the 2 remaining SNPs did not differ significantly between HYP and NT subjects (Table 3). Accordingly, further studies were focused on the effect of rs3754777 and rs1468326 on BP and hypertension.

**TABLE 3:**
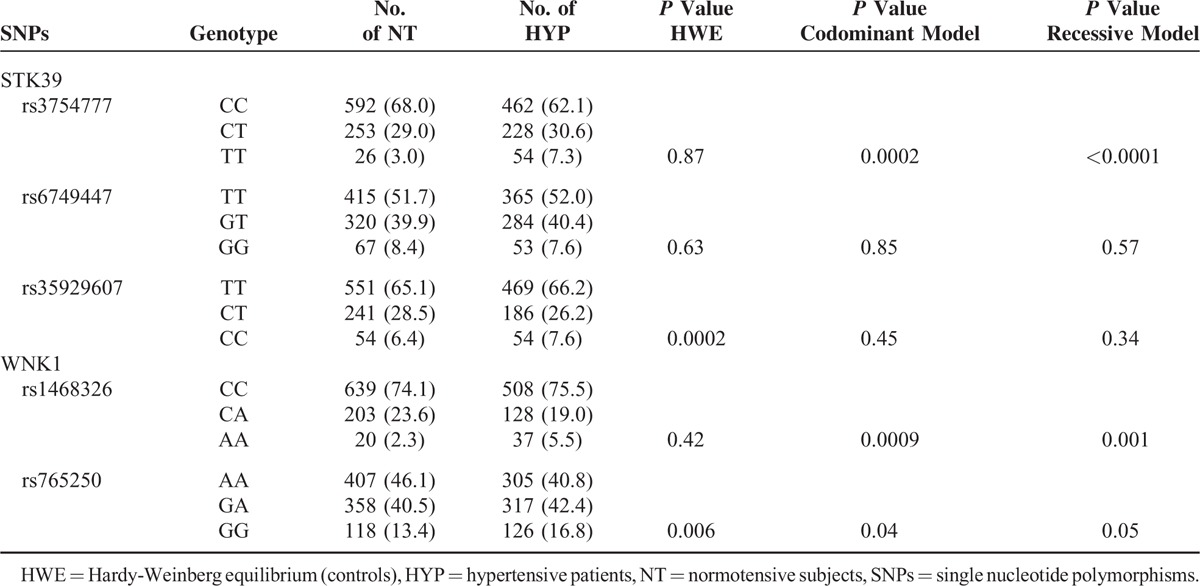
Association of Hypertension With Candidate Single Nucleotide Polymorphisms

### Association of SNP rs3754777 (STK39) With Hypertension and BP

**FIGURE 1:**
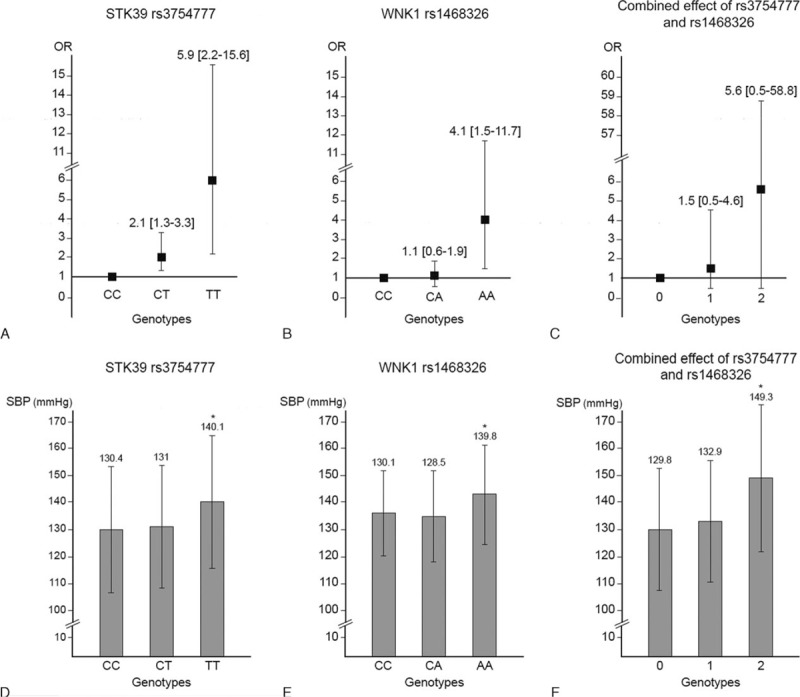
Odds ratios of being hypertensive (A–C) and mean SBP (D–F) according to genotype at rs3754777 and rs1468326. Odds ratios were adjusted for sex, age, body mass index, and total number of antihypertensive drug classes. The most frequent, wild-type genotype was used as the reference. In panels C and F, the numbers 0, 1, and 2 on the horizontal refer to homozygosity for the minor, “at-risk” allele at rs3754777 and rs1468326. ^∗^*P* < axis 0.05. SBP = systolic blood pressure level.

The distribution of genotypes of the rs3754777 SNP located within STK39 was significantly different either in an additive (*P* = 0.0002) or a recessive model (*P* < 0.0001), mainly due to a 2-fold higher prevalence of the rare TT genotype in hypertensive (7.3%) versus control subjects (3.0%) (Table 3). Compared with CC patients, patients harboring the minor T allele at the heterozygous or homozygous state had an OR of hypertension of 1.1 (95% confidence interval [CI]: 0.9–1.4) and 2.7 (95% CI: 1.6–4.3), respectively. The association was even more significant in a logistic regression model including age, sex, BMI, and total number of antihypertensive drug classes (OR CT vs CC: 2.1 [95% CI: 1.3–3.3]; TT vs CC: 5.9 [95% CI: 2.2–15.6]) (Figure 1A). Furthermore, in the whole data set including both cases and controls, mean office systolic BP was significantly higher in patients harboring the TT genotype compared with CT and CC patients (Figure 1D).

### Association of SNP rs1468326 (WNK1) With Hypertension and BP

Similarly, the distribution of genotypes of the WNK1 variant rs1468326 was significantly different in HYP and NT controls, both in an additive (*P* = 0.0009) and a recessive model (*P* = 0.001), with a 2-fold increase in prevalence of the rare AA genotype in HYP (5.5% vs 2.3%) (Table 3). Compared with CC patients, the OR of being hypertensive was significantly increased in AA (OR: 2.3 [95% CI: 1.3–4.1]) but not in CA patients (OR: 0.8 [95% CI: 0.6–1.0]), suggesting a recessive effect. The association was maintained, or even larger in a model including age, sex, BMI, and total number of antihypertensive drug classes (OR: 4.1 [95% CI: 1.5–11.7]) (Figure 1B). Finally, mean systolic BP was significantly higher in subjects harboring this genotype compared with CC and CA subjects (Figure 1E).

### Joint Effect of SNPs rs 3754777 (STK39) and rs1468326 (WNK1)

The distribution of genotypes in patients harboring 0, 1, or 2 of the at-risk genotypes (TT for rs3754777; AA for rs1468326) was also significantly different (*P* = 0.014) between hypertensive and normotensive subjects (normotensives: 95.6%, 4.0%, and 0.4% vs hypertensives: 92.1%, 6.8%, and 1.1%, respectively). Furthermore, in the whole data set, BP increased in a dose-dependent fashion according to the number of at-risk genotypes (Figure 1F). Along the same lines, the OR of being hypertensive tended to increase gradually according to the presence of 0, 1, or 2 at-risk alleles at the homozygous state (1 vs 0: OR: 1.7 [95% CI: 1.1–2.8]; 2 vs 0: OR: 3.1 [95% CI: 0.8–11.9]). The same trend was observed in the adjusted model (Figure 1C).

## DISCUSSION

We looked for an association between hypertension, BP, and genetic variants of *STK39* and *WNK1* in the BELHYPGEN case-control study, including predominantly severe hypertensive patients. A highly significant, consistent association was found for 2 of the 5 investigated SNPs, located respectively within the STK39 gene^6^ and upstream of the WNK1 promoter.^13^ Furthermore, despite the moderate sample size, our results suggest a synergistic effect of the variants. From a pathophysiological perspective, this fits with WNK1 being a regulator of SPAK, the product of *STK39*, which in turn activates the NCC.^7–10^

The association of the STK39 variant rs3754777 with hypertension is in agreement with the results of a GWAS published by Wang et al,^6^ one of the first “success stories” in the field of hypertension and a number of subsequent studies, recently summarized in a meta-analysis.^14^ Interestingly, while our analysis was limited to patients of Caucasian descent, in the latter, the effect of the STK39 variant was only found in Europeans and East-Asians, but not in Africans.^14^ In contrast, we were not able to replicate the associations with 2 other variants described in the initial GWAS,^6^ rs6749447 and rs35929607. In particular, our findings challenge the hypothesis that rs35929607 is the functional variant responsible for the effect of the “*STK39* locus” on BP, whereas rs3754777 is only a surrogate.^6^ Notably, this claim rests mostly on indirect evidence based on luciferase reporter gene constructs.^6^ The underlying truth may be much more complex, with several functional polymorphisms jointly accounting for the overall effect.^15^

An association between hypertension, BP, and variant rs1468326, located 3 kb from the promoter of *WNK1*, was already reported in severely hypertensive families from the BRIGHT study.^13^ Nevertheless, our study differs from the latter by the less convincing association with another WNK1 variant, rs765250.^16^ This discrepancy may reflect a lack of statistical power, as it was not detected in the first report of the BRIGHT study,^13^ but only in the second one, which included a much larger sample size.^16^ Notably, even in the latter, the association with hypertension per se was only borderline significant.^16^

Along with WNK4, WNK1 regulates numerous ion channels involved in sodium and potassium transport in the kidney and in various other epithelia.^7–10^ Mutations in *WNK1* are at the origin of pseudohypoaldosteronism type II (Gordon syndrome), characterized by hypertension and hyperkalemia.^17^ The role of the WNK-SPAK-NKCC2/NCC pathway is further highlighted by knockout mice models. In particular, heterozygous knockout mice for *WNK1*^18^ or *SPAK*^19^ exhibit a hypotensive phenotype. In addition, homozygous knockout mice, which completely lack SPAK expression, display hypokalemia, hypomagnesemia, and hypocalciuria (Gitelman-like syndrome) and impaired vasoconstriction.^19^ Finally, mice expressing a mutated SPAK, which cannot be activated by WNK isoforms, show decreased phosphorylation and expression of NKCC2 and NCC, and significantly lower, salt-dependent BP.^20^ On a lifelong basis, even subtle genetic changes leading to mild sodium and water accumulation may cause a significant alteration in sodium and water balance, fluid retention, and subsequent chronic hypertension.^21^

Although in complex traits such as hypertension, ORs are typically within the range of 1.1 to 1.3,^22^ and individual loci identified in GWAS account for 1 mm Hg BP at most,^23–25^ in our study, at-risk genotypes were associated with adjusted ORs of 4 to 6 and approximately 10 mm Hg higher mean BP compared with wild-type, admittedly with a large confidence interval. Although these relatively impressive results may be due to chance or some sort of “winner's curse” phenomenon,^26^ they may also result from inclusion of severe, difficult-to-treat hypertensive patients with early target organ damage, as expected in a tertiary referral cohort. In the latter, the contribution of genetics is expected to be higher than in late-onset, milder hypertension. Furthermore, the cases were compared with strictly normotensive, healthy controls. The effectiveness of using cases and controls at the extreme of the distribution for a continuous trait such as BP (the so-called “hypercontrols’ strategy”) is a known way to decrease noise and increase statistical power.^27,28^ Finally, statistical power may have been increased by limiting the analysis to patients of Caucasian descent.

Several limitations of our study should be acknowledged. The lack of association between rs35929607 and rs765250 and BP/hypertension should be interpreted with caution in view of the deviation from Hardy-Weinberg equilibrium, possibly reflecting population stratification, selection bias, unaccounted confounding factors, or, less probably, genotyping errors.^29,30^ Most patients (85%) were on antihypertensive treatment. Patients were recruited in tertiary referral centers, and thus even if replicated in other, similar cohorts, our findings may not be readily extrapolated to the overall hypertensive population. The control population was recruited in a single center of occupational health consultation including mostly health sector employees. As expected, the prevalence of women was high. Also, patients >65 years old were by definition excluded, leading to a substantial age difference between cases and controls. Still, adjustment for sex, age, BMI, and antihypertensive treatment did not change, and even reinforced our findings. Furthermore, BELHYPGEN is a unique cohort including patients from both the South and North of Belgium recruited in 6 of 7 Belgian medical faculties, and is thus likely representative of hypertensive patients seen at tertiary referral centers, providing reliable information on genetic epidemiology of this particular subset of at-risk hypertensive patients. Our sample size was of the same order of magnitude as in other studies with focus on *STK39*^6,14^ and *WNK1*,^13^ in which multiple SNPs were tested in either gene.

Our results need to be replicated in other clinical settings, including treatment-naive hypertensive patients in whom intermediary phenotypes, such as plasma renin activity, aldosterone plasma level, urinary sodium, and fractional sodium excretion, may be reliably assessed. Furthermore, the impact of variants located in other genes belonging to the WNK-SPAK-NKCC2/NCC pathway and other pathways related to water and sodium reabsorption may deserve to be jointly studied. Finally, unraveling the genetic epidemiology of sodium and water reabsorption in the kidney tubule may help to individualize antihypertensive treatment and identify new therapeutic targets.
